# Editorial: Biomarkers in the diagnosis, prognosis, and prediction of autoimmune and hereditary optic neuropathies

**DOI:** 10.3389/fneur.2023.1304227

**Published:** 2023-10-24

**Authors:** Chuan-bin Sun, Xiaolai Zhou, Hong Jiang, Huanfen Zhou

**Affiliations:** ^1^Eye Center, Second Affiliated Hospital of Zhejiang University School of Medicine, Hangzhou, China; ^2^State Key Laboratory of Ophthalmology, Zhongshan Ophthalmic Center, Sun Yat-sen University, Guangzhou, China; ^3^Department of Ophthalmology and Neurology, University of Miami Miller School of Medicine, Miami, FL, United States; ^4^Senior Department of Ophthalmology, The Chinese People's Liberation Army General Hospital, Beijing, China

**Keywords:** autoimmune optic neuritis, hereditary optic neuropathies, biomarkers, optical coherence tomography, optical coherence tomography angiography (OCTA), next-generation sequencing (NGS)

Autoimmune optic neuritis (ON) and hereditary optic neuropathies, such as Leber hereditary optic neuropathy (LHON), are both rare but sight-threatening ocular diseases. Their diagnosis and treatment in clinical practice are also challenging and continuously progressing. In the last decade, the detection and identification of aquaporin 4-IgG antibody (AQP4-Ab) and myelin oligodendrocyte glycoprotein-IgG antibody (MOG-Ab) have markedly promoted our understanding of the etiology, pathogenesis, classification, and prognosis of autoimmune ON. Technical advances in ophthalmic instruments including optical coherence tomography angiography (OCTA) and multiple color imaging and laboratory test methods such as next-generation sequencing have also updated the evaluation and analysis of the autoimmune ON and hereditary optic neuropathies. It is therefore of high interest to collect and summarize the emerging findings in studies about *Biomarkers in the diagnosis, prognosis, and prediction of autoimmune and hereditary optic neuropathies*.

This Research Topic aimed to collect the most recent progressions in basic and clinical investigations about humoral, genetic, and imaging biomarkers for autoimmune and hereditary optic neuropathies, especially the detection and identification of novel biomarkers showing probability in clinical translation.

Early detection of retinal nerve fiber loss is of great importance to evaluate visual function defects in optic neuropathies such as ON, anterior ischemic optic neuropathy (AION), and glaucoma. However, the sensitivity of OCT in detecting retinal nerve fiber layer (RNFL) loss in early-stage optic atrophy is still not satisfactory due to data overlap between patients with early-stage optic atrophy and normal controls. In one study, Cheng et al. evaluated the value of multicolor imaging compared to conventional color fundus photography in detecting RNFL defects in LHON cases and revealed that multicolor imaging, particularly green reflectance and green–blue-enhanced imaging, was more efficient in detecting RNFL defects than color fundus photography, indicating that multicolor imaging may be a useful and inexpensive alternative to detect or monitor RNFL defects.

Although AQP4-Ab and MOG-Ab have been reported in part of ON cases, most cases with ON showed negative test results for AQP4-Ab and MOG-Ab. Hence, there may exist new biomarkers currently unknown in cases with ON. In another study, Sun et al. reported a case of *Orientia tsutsugamushi* infection-related ON using metagenomic next-generation sequencing. Unlike those instances of ON reported in the literature that usually presented after systemic manifestations of *Orientia tsutsugamushi*, such as fever, eschar, lymphadenopathy, rash, and other flu-like signs, their study revealed that ON might manifest as the initial presentation of *Orientia tsutsugamushi* infection, which makes the diagnosis much more challenging. For this case, detecting *Orientia tsutsugamushi* by metagenomic next-generation sequencing is of great value for confirming the etiology of ON and subsequent proper therapy to save the patient's sight. Moreover, in this case, serum tests for AQP 4-Ab and MOG-Ab were both negative, but they were both positive for anticardiolipin IgM and beta-2-glycoprotein-I IgM, indicating that pathogen-induced anticardiolipin antibody production might be involved in the induction of ON. Similar findings were also reported in previous investigations on viral and parasite infection-related ON.

The current staging of AION is mainly based on ophthalmic manifestations. Previous pilot investigations on the staging of AION based on OCTA were mostly retrospective studies and lacked longitudinal observations of blood flow changes of the optic disk. In another study, Xiao and Sun evaluated the longitudinal changes in the blood flow of the optic disk in patients with non-arteritic AION using OCTA. They revealed that the radial peripapillary capillary density, the blood flow indicator of the optic disk, was significantly reduced during the acute-staged (≤3 weeks) and subacute-staged (≥3 weeks, <8 weeks) NAION and kept stabilized 8 weeks after onset. The areas of decreased radial peripapillary capillary density, visual field defect, and macular ganglion cell complex loss showed good spacial correspondence in their cases, which indicates that OCTA is a good method for dynamically monitoring the changes in the blood flow of the optic disk.

OCT and OCTA are emerging methods to evaluate the severity of demyelinating diseases of the central nervous system. In another study, Li et al. assessed the relationship among the changes in macular and peripapillary vessel density, the existence of microcystic macular edema (MME), and the visual impairment and disease disability in neuromyelitis optica spectrum disorder (NMOSD) patients using OCTA. They found that the existence of MME was related to worse visual impairment and disability in NMOSD patients. A decrease in the superficial retinal capillary plexus and radial peripapillary capillary density was observed in NMOSD MME eyes and related to worse best-corrected visual acuity and Expanded Disability Status Scale scores, which implies that OCTA may play a role in predicting the severity of systemic disability in NMOSD cases.

In summary, this Research Topic provided updated insights into the progress in the diagnosis and evaluation of ON and hereditary optic neuropathy. The studies collected in this Research Topic revealed that multicolor imaging is a useful and inexpensive alternative to detect or monitor RNFL defects, the pathogen-induced anticardiolipin antibody might play a role in the induction of ON, OCTA could dynamically monitor changes in the blood flow of the optic disk, and OCTA might help to predict the severity of systemic disability in NMOSD cases. Nevertheless, more investigations are still needed to further explore the pathogenesis of demyelinating ON, such as new biomarker detection, and of hereditary optic neuropathy, such as new causative gene mutation points detection. Moreover, better evaluation methods are also needed to assess the value of some uncommon clinical findings in emerging clinical trials for hereditary optic neuropathies. For example, how do we explain visual improvements in both eyes even when gene therapy is performed in only one eye in LHON clinical trials?

## Author contributions

C-bS: Writing – original draft, Writing – review & editing. XZ: Writing – original draft, Writing – review & editing. HJ: Writing – original draft, Writing – review & editing. HZ: Writing – original draft, Writing – review & editing.

